# Pillared Carbon Membranes Derived from Cardo Polymers

**DOI:** 10.3390/nano13162291

**Published:** 2023-08-09

**Authors:** Masoumeh Tajik, Syed Fahad Bin Haque, Edson V. Perez, Juan P. Vizuet, Hamid Reza Firouzi, Kenneth J. Balkus, Inga H. Musselman, John P. Ferraris

**Affiliations:** Department of Chemistry and Biochemistry, The University of Texas at Dallas, 800 W. Campbell Road, Richardson, TX 75080-3021, USA; masoumeh.tajikasl@utdallas.edu (M.T.); syedfahadbin.haque@utdallas.edu (S.F.B.H.); edson.perez@utdallas.edu (E.V.P.); juan.vizuet@utdallas.edu (J.P.V.); hamidreza.firouzi@utdallas.edu (H.R.F.); kenneth.balkus@utdallas.edu (K.J.B.J.); inga.musselman@utdallas.edu (I.H.M.)

**Keywords:** carbon molecular sieve membranes, pillaring by metal nanoparticles, stability against physical aging

## Abstract

Carbon molecular sieve membranes (CMSMs) were prepared by carbonizing the high free volume polyimide BTDA-BAF that is obtained from the reaction of benzophenone-3,3′,4,4′-tetracarboxylic dianhydride (BTDA) and 9,9-bis(4-aminophenyl) fluorene (BAF). The bulky cardo groups prevented a tight packing and rotation of the chains that leads to high permeabilities of their CMSMs. The incorporation of metal–organic polyhedra 18 (MOP-18, a copper-based MOP) in the BTDA-BAF polymer before pyrolysis at 550 °C prevented the collapse of the pores and the aging of the CMSMs. It was found that upon decomposition of MOP-18, a distribution of copper nanoparticles minimized the collapse of the graphitic sheets that formed the micropores and mesopores in the CMSM. The pillared CMSMs displayed CO_2_ and CH_4_ permeabilities of 12,729 and 659 Barrer, respectively, with a CO_2_/CH_4_ selectivity of 19.3 after 3 weeks of aging. The permselectivity properties of these membranes was determined to be at the 2019 Robeson upper bound. In contrast, the CMSMs from pure BTDA-BAF aged three times faster than the CMSMs from MOP-18/BTDA-BAF and exhibited lower CO_2_ and CH_4_ permeabilities of 5337 and 573 Barrer, respectively, with a CO_2_/CH_4_ selectivity of 9.3. The non-pillared CMSMs performed below the upper bound.

## 1. Introduction

**Figure 1:**
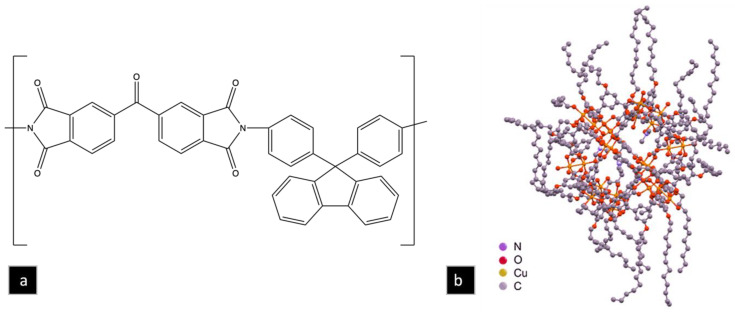
Structure of (**a**) BTDA-BAF and (**b**) MOP-18.

Carbon molecular sieve membranes (CMSMs) containing micropores (0.7–2.0 nm) and ultramicropores (<0.7 nm) have the potential to surpass the Robeson upper-bound for the separation of gas mixtures such as CO_2_/CH_4_ [1]. This is possible because the micropores in these membranes provide a high gas flux, while their ultramicropores perform molecular sieving. The choice of the CMSM polymer precursor is important because it affects the pore structure of the membrane and, ultimately, its performance. Polyimides are good precursors due to their good chemical, thermal and mechanical properties [2] but chain packing and segmental motion [3] affect their separation properties. The introduction of bulky groups in the backbone, such as a cardo moiety (Figure 1a), can significantly increase the free volume in the polymer [3,4] and in the resulting CMSM by disrupting the packing of the polymer chains. Several cardo polymers have been reported for CO_2_ separations [5,6,7,8,9,10,11,12,13,14,15,16,17,18]. A series of cardo polymers containing bis(4-aminophenyl) fluorene, which show high thermal stability and solubility, have been reviewed by Korshak [5]. Yahaya et al. studied a random co-polyimide membrane of 6FDA-durene/cardo with different ratios of the cardo moiety in the backbone. In this study, the co-polyimide of 6FDA-durene/cardo (3:1) was stable at pressures of 21 bar and up to 20% H_2_S in the feed gas [17]. In another study, Hu et al. compared the permselectivity of CMSMs derived from the ODPA-FDA polyimide with Matrimid (Figure S1 in Supplementary Materials). The results showed an increase in the T_g_ and *d*-spacing in the ODPA-FDA polymer due to the addition of the bulky aromatic group. This was also reflected in the free volumes determined via PALS (0.678% for ODPA-FDA compared to 0.52% for Matrimid). The increase in free volume is even more impressive when one considers that the dianhydride of ODPA is smaller than that of Matrimid [18]. Sun et al. also reported that the fractional free volumes calculated via molecular simulation were 29.4% for PIM-1 and 32.7% for cardo-PIM-1, an 11% increase in fractional free volume [19]. These studies suggest that the fractional free volume of BTDA-BAF would be higher than that of Matrimid (calculated FFV of 19% [20]) but lower than that of PIM-1.

The rigid micropores of CMSMs generally results with better permeability and selectivity than the less rigid ones in polymer membranes [21,22,23]. Although CMSMs display improved permselectivity [24,25], they are subject to physical aging due to the densification of the CMSM from the collapsing of the pores [26,27]. The CMSM densification results from the rearrangement of the graphitic sheets to reach an equilibrium state from the as-formed non-equilibrium state. Strategies aimed at minimizing aging include carbonization at higher temperatures, crosslinking, and pillaring. Carbonization at high temperature can reduce the physical aging of CMSM due to the narrowing of the pore structure but results in a decline in permeability and mechanical stability [28,29]. Crosslinking imparts polymer rigidity and plasticization-resistant properties with high permselectivity [30,31,32,33,34,35,36] and reduction in physical aging [37,38,39,40] but is limited to the availability of functional groups in the precursor. Nakagawa et al. reported the control of micropores via pillaring in a carbonized ion exchange resin [41], and, more recently, pillaring of CMSMs from the pyrolysis of MOP-18/PIM-1 mixed matrix membranes (MMMs) was reported by Cosey et al. [42]. In this study, copper nanoparticles were created from the pyrolysis of the soluble copper-based metal–organic polyhedra (MOP-18, Figure 1b) dispersed in PIM-1. The copper nanoparticles dispersed in the CMSMs acted like pillars that prevented the collapse of the micropores and densification of the membranes during the carbonization. In this study, 40 wt% MOP-18 loading was found to be an optimum concentration to prevent aging, which was used as a starting point in the present work.

In this work, CMSMs from the contorted cardo polyimide BTDA-BAF and MOP-18/BTDA-BAF MMMs with FFV higher than that of Matrimid but lower than that of PIM-1 were prepared. The permselectivity for CO_2_/CH_4_ and physical aging of the CMSMS with and without the presence of copper nanoparticles from MOP-18 was studied.

## 2. Experimental

### 2.1. Materials

Ethyl acetate, sodium bicarbonate, potassium hydroxide, ethanol, tetrachloroethane, *N*,*N*-dimethylformamide, concentrated sulfuric acid, 1-methyl-2-pyrrolidone (NMP, 99.8%), *N*,*N*-dimethylacetamide (DMAc, anhydrous), dimethyl sulfoxide (DMSO)_-*d*6_, and benzophenone-3,3′,4,4′-tetracarboxylic dianhydride (BTDA) were purchased from Sigma Aldrich, St. Louis, MO, USA. 9,9-bis(4-aminophenyl) fluorene (BAF, >98%) was purchased from TCI, Montgomeryville, PA, USA. Xylene (98.5%) was purchased from EMD, Burlington, MA, USA. 1-iodododecane and 5-hydroxyisophthalic acid were purchased from ThermoFisher Scientific, Waltham, MA, USA. Copper acetate monohydrate was purchased from Alfa Aesar, TheromoFisher Scientific, Waltham, MA, USA. Hydrochloric acid was purchased from J.T. Baker, Phillipsburg, NJ, USA.

### 2.2. Polymer Synthesis

The synthesis of BTDA-BAF (Figure 1a) was carried out via thermal imidization. All glassware was dried at 120 °C for 24 h. A 50 mL graduated constant pressure additional funnel, and a condenser were fitted to the top of the two-neck round bottom flask (100 mL). Dried 4A molecular sieves were added to the graduated additional funnel to trap water produced during the reaction. The apparatus was sealed with two rubber septa on top of the condenser and one of the necks in the round bottom flask. To minimize the amount of water in the reaction mixture, the apparatus was evacuated three times and purged with nitrogen to provide an inert atmosphere. Then, 20 wt% solutions of BTDA (2.50 g, 7.75 mmol) and BAF (2.70 g, 7.75 mmol) in NMP were prepared separately. Next, the BAF solution was transferred to the round bottom flask, and the BTDA solution was added dropwise. The mixture was allowed to stir at room temperature for 1 d. Then, 12 mL of xylene was added to the viscous polyamic acid solution to make an azeotropic mixture for water removal from the system and the temperature increased to 150 °C to complete the imidization. The mixture was then stirred for 1 d at 150 °C after which the polyimide was precipitated into 300 mL of methanol. The precipitate was filtered and purified using Soxhlet extraction with methanol for 48 h. The purified polymer was then dried under vacuum at 200 °C for 24 h (Yield: 92%; Mw: 36;000 Da; PDI: 2.4 by GPC; 500 MHz ^1^H NMR in CDCl_3_ is shown in Figure S2 in the Supporting Materials). MOP-18 was prepared following the procedure reported in previous studies [43,44].

### 2.3. Polymer Precursor Preparation

BTDA-BAF membranes were prepared from a 15 wt% solution in tetrachloroethane by stirring for 1 d at room temperature. The 40% (*w*/*w*) MOP-18/BTDA-BAF MMM solution was prepared by dissolving separately 0.5 g of BTDA-BAF in 3.33 g of tetrachloroethane and 0.2 g of MOP-18 in 2 g of tetrachloroethane. The solutions were stirred for 1 d, combined, and stirred for 1 d. The MOP-18 loading was based on the reported optimal concentration for the stabilization of PIM-1 [42]. The solutions were cast inside a laminar flow fume hood on a glass substrate using a Sheen 1133N, automatic applicator with an adjustable blade set to 500–700 mm, Sheen Instruments Ltd., Kingston, UK. After drying for 6 h in a custom-built drying table at RT under N_2_ purge, the flat membranes were peeled off from the glass substrate (Figure S3a in Supplementary Materials) and annealed at 150 °C for 1 d in a vacuum oven. The membranes’ thicknesses were measured by scanning electron microcopy (SEM) and ranged from 50 mm to 60 mm (Figure S4 in Supplementary Materials).

### 2.4. CMSM Fabrication

The flat membranes were placed on a graphite plate inside a three-inch quartz tube in a three-zone tube furnace (MSI-1200X-III) equipped with a PID programmable temperature controller (Omega Engineering, Inc., Norwalk, CT, USA, model CN1507TC). The furnace was evacuated for 1 h and then purged continuously with ultra-high purity N_2_ at 200 cm^3^/min for the pyrolysis duration. CMSMs were pyrolyzed using the temperature protocol shown below [26]:

1. 20 to 250 °C at a rate of 13 °C/min;
2. 250 to 535 °C at a rate of 3.85 °C/min;
3. 535 to 550 °C at a rate of 0.25 °C/min;
4. 0 h soak time at 550 °C.

## 3. Characterization

### 3.1. Polymer

The molecular weight of the polymer was determined via size exclusion chromatography (SEC) using a Viscotek VE 3580 system equipped with a Viscotek Column T6000M connected to refractive index (RI), light scattering, and viscosity detectors, Malvern Panalytical, Malvern, UK The SEC solvent/sample module (GPCmax) used HPLC-grade tetrahydrofuran as the eluent and was calibrated with polystyrene standards. ^1^HNMR spectra were obtained using a Bruker III 500 MHz spectrometer. Samples were prepared in CDCl_3_ and DMSO_-*d*6_ with TMS as the internal standard.

### 3.2. Membranes

Thermal gravimetric analyses (TGA) on 50 mg samples were carried out using a Perkin Elmer Pyris-1 TGA (Perkin Elmer, Shelton, CT, USA), thermogravimetric analyzer under UHP N_2_ flow of 20 mL/min and a ramp rate of 10 °C/min.

Attenuated total reflectance–Fourier transform infrared spectra (ATR-FTIR) were acquired using a Nicolet 360 FTIR instrument, Thermo Fisher Scientific, Waltham, MA, USA. A total of 64 scans with a 2 cm^−1^ resolution were acquired.

A Mettler Toledo TGA/DSC 1 equipped with a quartz capillary interface to a quadrupole mass spectrometer, Pfeiffer ThermoStar, Pfeiffer Vacuum, Aβlar, Germany (300 a.m.u range), was used to acquire thermogravimetric/mass spectra from the thermal decomposition of MOP-18 in argon. The argon flow rate was set to 50 mL/min and the temperature programmed as follows: settling at 25 °C for 30 min, then ramp to 250 °C at 13 °C/min, to 535 °C at 3.85 °C/min, and to 550 °C at 0.25 °C/min. The capillary interface was heated to 190 °C and the mass spectrometer was operated at 3 × 10^−6^ mbar with the SE detector set to 980 V.

### 3.3. CMSM

X-ray diffraction analyses of CMSM samples with and without copper particles from MOP-18 pyrolysis were obtained using a Rigaku Ultima III diffractometer with Cu Kα X-ray radiation (1°/min scan rate, 0.2° step). Raman spectra were collected using a DXR Raman spectrophotometer equipped with a 532 nm laser. N_2_ and CO_2_ adsorption–desorption data were collected using a Micromeritics ASAP 2020 gas adsorption apparatus, Micromiretics, Norcross, GA, USA. Calculation of surface area of the CMSM was performed using the Brunauer–Emmett–Teller (BET) method. Pore size distribution was analyzed using the two-dimensional nonlocal density functional theory (2D-NLDFT) technique implemented in the SAIEUS software, ver 3.0 (Thermo Fisher Scientific, Waltham, MA, USA) A JEOL 1400+ transmission electron microscope (TEM) operated at 200 kV was used to image the copper particles in the CMSMs. The copper particle size was measured using ImageJ software, ver 1.53 (Pfeiffer Vacuum, Ablar, Germany). X-ray photoelectron spectra (XPS) were taken using a PHI VersaProbeII Scanning XPS Microprobe (Physical Electronics Inc., Chanhassen, MN, USA) equipped with an Al-K_α_-X-ray source (E = 1486.7 eV). The pressure during the measurement was kept lower than 5 × 10^−10^ mbar. Spectra were collected using 23.5 eV pass energy at a resolution of 0.2 eV. All spectra were collected using charge compensation using an electron beam incident to the surface.

### 3.4. Gas Permeability Testing

The pure gas permeability of each membrane was obtained at 35 °C and 2 atm using custom-built permeameters with a LabVIEW 16.0 software interface (National Instruments) [45,46]. Typically, a flat membrane with known thickness and area was mounted into the permeation cell and degassed for at least 12 h. To ensure accurate permeability measurements, a leak test was performed before each experiment and measured to be less than 0.013 mbar/h. During the experiments, the upstream side of the permeation cell was pressurized to 2 atm with either CH_4_ or CO_2_. The upstream and downstream sides were degassed for 2 h between runs and 4 h before switching gases. The apparatus was also degassed 24 h between each set of 4 runs per gas. Equation (1) shows the relation between the permeability of gas (P_i_) in Barrer, gas flux (n_i_), membrane thickness (*l*), membrane exposed area (A), and transmembrane partial pressure difference (∆p_i_). The permeability was calculated using the slope of downstream pressure versus time curve at the steady.

(1) $$P_{i}=\frac{\left(ni)(l\right)}{\left(∆pi\right)}$$

The ideal selectivity α_A/B_ for two penetrants is the ratio of the component permeabilities.

### 3.5. Aging Experiments

The membranes were kept in the permeation cell for the entire duration of the aging study. The aging studies for both the pure BTDA-BAF and the Cu-pillared BTDA-BAF CMSMs lasted for 21 d. The aging experiments were performed by evacuating the upstream and downstream between runs and gases. During the experiments, the upstream was pressurized to 2 atm with either CO_2_ or CH_4,_ and the downstream pressure recorded.

## 4. Result and Discussion

### 4.1. Thermal Analysis (TGA, TGA-MS, DSC) of Membranes

**Figure 2:**
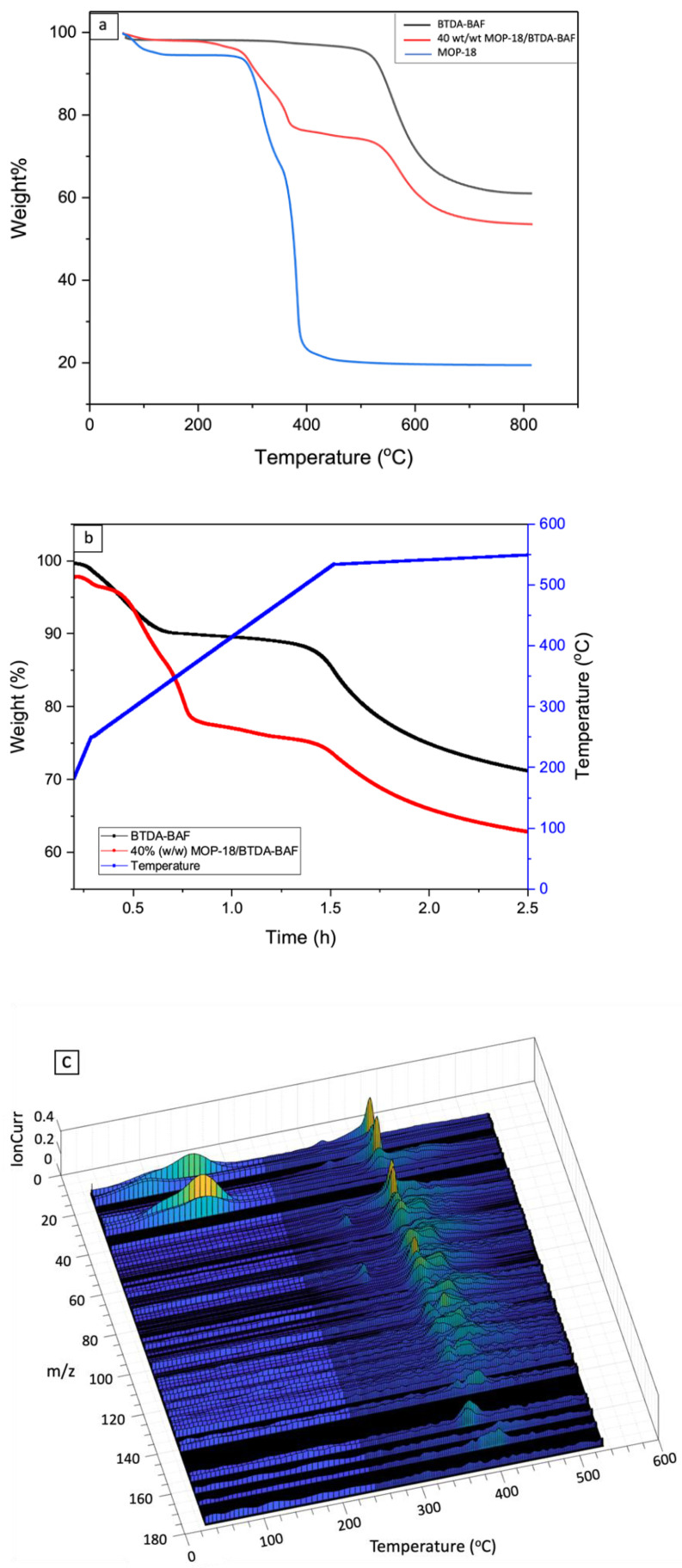
(**a**) TGA plots for pristine BTDA-BAF (black), 40% (*w*/*w*) MOP-18/BTDA-BAF (red) and MOP-18 (blue); (**b**) TGA following the carbonization protocol for BTDA-BAF (black), 40% (*w*/*w*) MOP-18/BTDA-BAF; and (**c**) TGA-MS spectra of MOP-18 following the carbonization protocol up to 550 °C.

Thermal gravimetric analysis (TGA) was used to analyze the thermal degradation of pristine BTDA-BAF, MOP-18, and 40% (*w*/*w*) MOP-18/BTDA-BAF. A 20% weight loss at 575 °C was observed for BTDA-BAF and a weight loss of 22% at 380 °C for the decomposition of MOP-18 in the MMM. Thermal analysis of 40% (*w*/*w*) MOP-18/BTDA-BAF showed a 40% weight loss around 575 °C (Figure 2).

The thermal stability of the polymer precursor was also examined to determine the degree of carbonization. TGA shows that 61% of the polymer precursor of BTDA-BAF, 53% of 40% (*w*/*w*) MOP-18/BTDA-BAF and 21% of MOP-18 remain after the pyrolysis. Additionally, TGA programmed with the carbonization protocol showed that MOP-18 decomposed at 350 °C with the remaining weight loss due to the BTDA-BAF. The remaining weight at 550 °C for the 40% (*w*/*w*) MOP-18/BTDA-BAF was 64% and 72% for the BTDA-BAF, which indicates 8% is from MOP-18, from which 5% is copper and 3% carbon.

The high glass transition temperature of 384 °C of the pure BTDA-BAF suggests the cardo moiety restricts the rotational mobility of the polymer backbone and hinders the chain packing (Figure S5 in Supplementary Materials). For the MOP-18/BTD-BAF MMM, the glass transition temperature increased to 436 °C, suggesting further restriction of the polymer chain movement upon incorporation of MOP-18, which is consistent with the reported increase in modulus in other MOP-18 containing MMMs [44].

Figure 2a shows that MOP-18 releases 10% of material up to 200 °C, which is attributed to adsorbed guest molecules from the synthesis (e.g., cleaning solvents) and adsorbed water. A second and third weight loss are observed from 300 °C to 380 °C, which are associated with the degradation of the organic linker. The species observed in the mass spectrum in this temperature range include CO_2_ (*m*/*z* 44) at 300 °C and 380 °C and C_m_H_n_ fragments with *m*/*z* values of up to 168 (Figure 2c) in the 380 °C to 420 °C temperature range (Figure 2c). Similar decomposition temperatures for MOP-18 were also observed by Furukawa et al. [43]. Considering that above 200 °C the remaining weight is from pure, free of impurities MOP-18, then it can be concluded that the second and third weight losses represent 83.3% of the remaining weight and the 16.7% of the solid CuO that remains at the end of the pyrolysis. These values are very close to the theoretical amounts of 84.6% of linker and 15.4% of copper present in the structure of MOP-18 before decomposition. During decomposition, however, the amount of oxygen retained by copper as CuO at the end of the pyrolysis directly impacts the amount of oxygen present in the linker, therefore lowering the weight to 83.3%. The weight of CuO drops from the theoretical 19.8% (if all oxygen reacted with copper to produce CuO) to the measured 16.7%.

The decomposition of MOP-18 in the temperature ranges from 300 °C to 420 °C may have a significant impact on the properties of the CMSM. Since the decomposition occurs at the polymer’s T_g_, it is plausible that the released linker is acting as a pore generator during the formation of the CMSM since it is present in a significant amount in the polymer precursor. If the porosity of the polymer precursor was indeed increased before the formation of the graphitic domains, then the porosity of the resulting CMSM may have also been increased.

### 4.2. Spectroscopic Characterization of Mixed Matrix and Carbon Membranes

**Figure 3:**
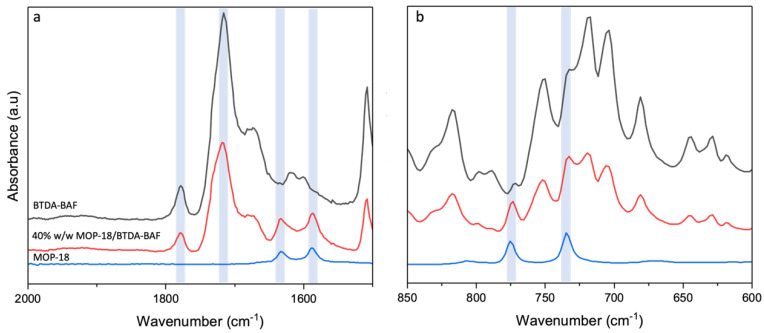
ATR-FTIR of BTDA-BAF (black), 40% (*w*/*w*) MOP-18/BTDA-BAF (red), and MOP-18 (blue): (**a**) C=O and C=C alkene stretch, and (**b**) C-O stretch.

ATR-FTIR was used to confirm the incorporation of MOP-18 into the polymeric membrane of BTDA-BAF. The characteristic imide stretching vibrations for C=O were observed at 1714 cm^−1^ (symmetric) and 1778 cm^−1^ (asymmetric) for the pristine polymer and MMM (Figure 3a). The two peaks associated with MOP-18 appeared at 1587 cm^−1^ and 1633 cm^−1^, which are attributed to the C=C stretching (Figure 3a). The C-O stretch at 734 cm^−1^ also indicated the presence of MOP-18 in the polymer matrix.

### 4.3. Transmission Electron Microscopy (TEM)

**Figure 4:**
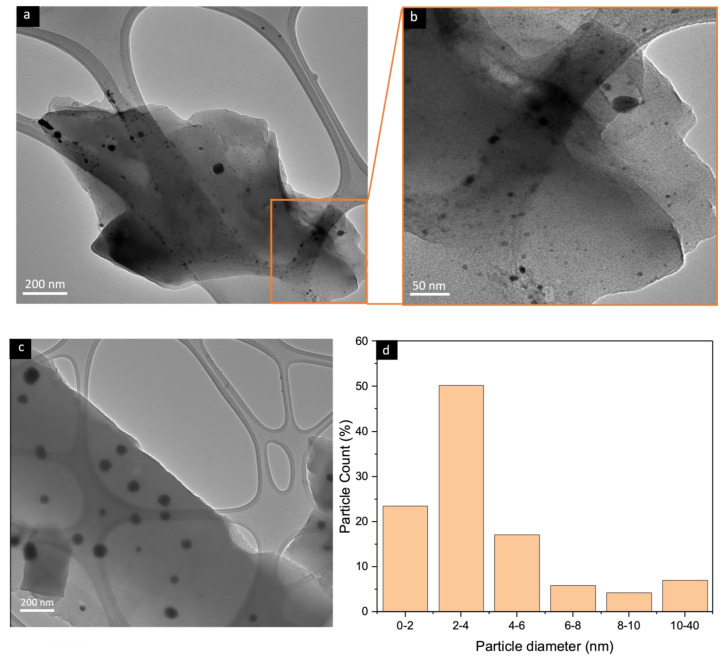
(**a**–**c**) TEM images of a 40% (*w*/*w*) MOP-18/BTDA-BAF CMSM and (**d**) the corresponding histogram of particle size distribution.

Figure 4 shows the TEM images of the CMSM from the pyrolysis of a 40% (*w*/*w*) MOP-18/BTDA-BAF MMM. The figure shows that the majority of the particles (70%) have sizes between 1 and 4 nm and have the potential of retarding the collapse of the micropores and mesopores in the CMSM. The membranes pyrolyzed with 0 h of soaking time exhibited a higher number of copper particles with sizes smaller than 4 nm due to the lower tendency of the particles to sinter with a decreased pyrolysis time [47]. Ostwald ripening is a thermodynamic process that can produce larger particles at the expense of smaller ones. The growth of the larger copper particles is time-dependent, with longer soaking time providing more chances for the nanoparticles to sinter. Yeshchenko et al. studied the size-dependent melting of copper nanoparticles and found that the melting of the nanoparticles was hindered for particles approaching 20 nm. The surface melting occurs at temperatures much lower than the melting of the bulk but it can jump to the melting point of bulk copper once the particle size becomes greater than 20 nm [48]. Pyrolysis at 550 °C and soaking times of 0 h (550 °C-0 h) results in particle sizes smaller than 20 nm with minimum sintering of the nanoparticles [47].

### 4.4. XRD, Raman, and X-ray Photoelectron Spectroscopic Analysis

**Figure 5:**
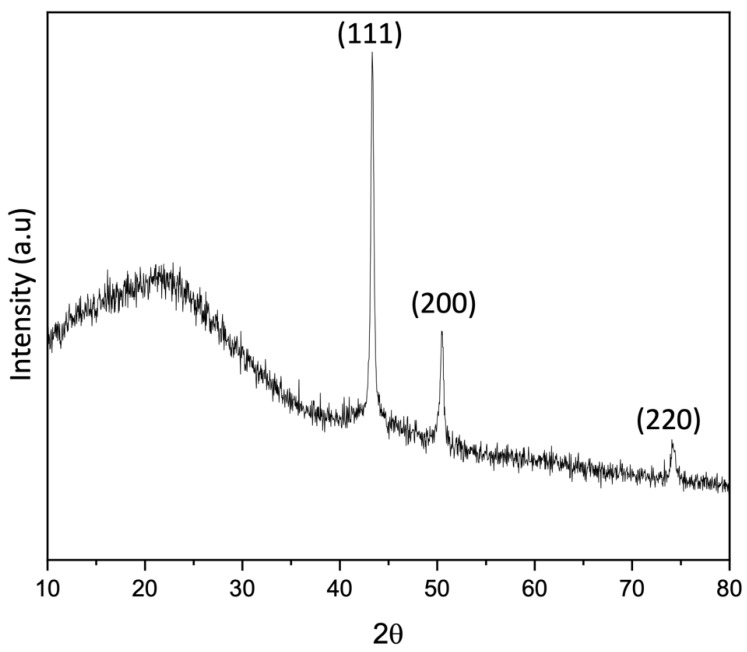
X-ray diffraction pattern of 40% (*w*/*w*) MOP-18/BTDA-BAF CMSM.

The three peaks at 2θ° of 43.28°, 50.50°, and 74.24° (JCPDS No. 04-0836) in XRD (Figure 5) are related to the 111, 200, and 220 reflections of copper metal, confirming the in situ formation of copper nanoparticles upon thermal degradation of MOP-18 starting at 300 °C. The porosity and gas adsorption properties of MOP-18, however, are lost when the MOP-18/BTDA-BAF is pyrolyzed.

Raman spectroscopy was used to estimate the degree of graphitization. The Raman spectrum of graphite is defined by the presence of two sharp peaks. The peak around 1580–1600 cm^−1^ known as the G-band is associated with ordered graphitized carbon (sp^2^), while the peak around 1350 cm^−1^ known as the D-band is related to disordered amorphous carbonaceous species (sp^3^) [49,50].

**Figure 6:**
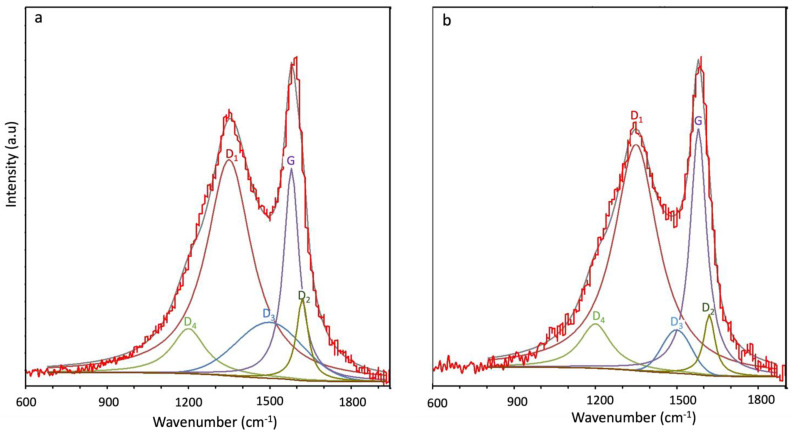
Raman spectra of (**a**) BTDA-BAF and, (**b**) 40% (*w*/*w*) MOP-18/BTDA-BAF CMSM.

The Raman spectra of CMSM of BTDA-BAF and 40% (*w*/*w*) MOP-18/ BTDA-BAF at 550 °C-0 h are shown in Figure 6. The I_D_/I_G_ ratio of the pyrolyzed BTDA-BAF is 1.02, while the I_D_/I_G_ of the pyrolyzed 40% (*w*/*w*) MOP-18/BTDA-BAF is 0.92 (Table 1). The Raman shift to lower I_D_/I_G_ was reported in MOP-18/PIM-1 as well [42]. The I_D_/I_G_ ratio of pyrolyzed MOP-18 at 550 °C is 0.72 [42], and the lower I_D_/I_G_ ratio in the pyrolyzed MMM could be due to the carbon associated with the MOP-18 [42]. Raman spectroscopy also gives information about the graphite crystallite size (L_a_, Å) calculated using Equation (2), which is proportional to the I_D_/I_G_ ratio. The increase in the graphite crystallite size for the Cu-pillared carbon membrane (L_a_ = 47.82), compared to the non-pillared (L_a_ = 43.13), correlates to a decline in terminal sp^3^ carbon sites [42]. Reducing the number of sp^3^ carbon sites means less distortion can be expected in the CMSM with larger graphite crystallite size.

La = 44(I_D_/I_G_)^−1^ (2)

The smaller I_D_/I_G_ ratio is associated with higher graphitic content and smaller defect density [51] and indicates a high graphitization level when the crystal size is larger than 20 Å [49]. The graphite crystallite size increased from 43 Å in BTDA-BAF to 47 Å in 40% (*w*/*w*) MOP-18/BTDA-BAF. The larger graphitic crystal size will impede rearrangement through time in CMSM [42]. The deconvolution into five bands was performed to best fit the Raman for CMSMs, as identified in Figure 6 and Table S1 [52]. The D_2_ band appeared at 1620 cm^−1^, associated with the graphitic lattice vibration (isolated graphene layers) versus the G band (stacked graphene layers) [53,54]. The D_2_ band, also known as the disordered graphitic sheet, is higher in the CMSM of BTDA-BAF compared to 40% (*w*/*w*) MOP-18/BTDA-BAF suggesting the larger portion of isolated graphene layers in the BTDA-BAF which are susceptible to rearrangement during aging. On the other hand, the larger graphite crystallite size and more stacked graphene layers indicate more ordered structures which are less prone to rearrangement in the 40% (*w*/*w*) MOP-18/BTDA-BAF. Rearrangement of CMSM is plausible over time in those with higher ID/IG ratio and smaller unconnected domains.

**Figure 7:**
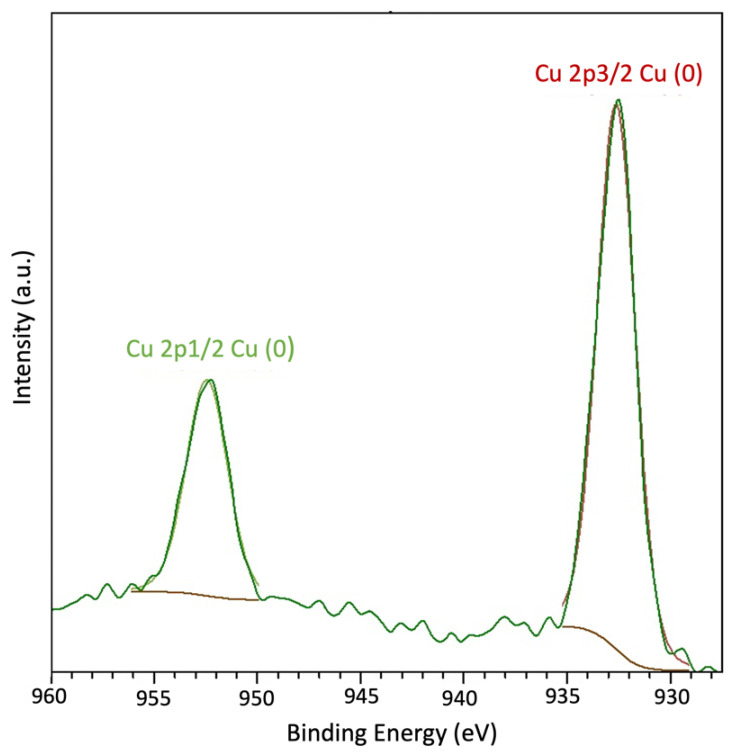
XPS spectra of 40% (*w*/*w*) MOP-18/BTDA-BAF CMSM for Cu 2p.

**Figure 8:**
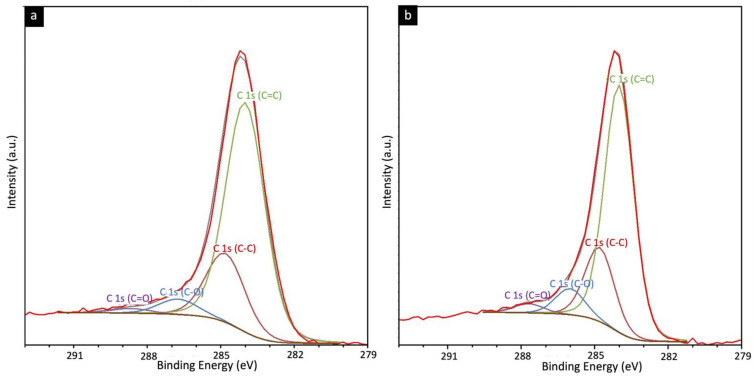
XPS spectra of (**a**) BTDA-BAF and, (**b**) 40% (*w*/*w*) MOP-18/BTDA-BAF CMSM for C 1s.

X-ray photoelectron spectroscopy was used on both carbon membranes with and without MOP-18 to identify surface functionality (Figure S6). In Figure 7, XPS analysis of a 40% (*w*/*w*) MOP-18/BTDA-BAF-derived CMSM showed two peaks corresponding to copper metal at 932 eV and 952 eV. The lack of a shakeup satellite peak related to the CuO around 945 eV confirms the formation of copper metal. The deconvoluted C1s spectrum in Figure 8 shows 284.0 eV and 284.8 eV peaks corresponding to the C=C (sp^2^) and C-C (sp^3^). The presence of small traces of ketone and carboxylate functional groups was noted in the XPS. The higher percentage of C-O and C=O bands in MOP-18/BTDA-BAF compared to the BTDA-BAF is due to the contribution of the carboxylic group in the MOP-18.

### 4.5. Gas Adsorption and Pore Size Distribution Analysis

Physical aging, a thermodynamically driven process, leads to the formation of denser membranes via the collapsing of the pore structure. A reduction in the number of micropores and ultramicropores over time results in a decrease in permeability and an increase in selectivity [27,55]. During pyrolysis, the polyimide chains tend to be aromatized along with the elimination of methyl and carbonyl groups. When the functional groups are removed, the slits between the aromatized strands, known as ultramicropores, are developed. Hays et al. discussed earlier that ultramicropores might control physical aging in CMSMs [55]. Tightening these pores will impact the diffusion of a larger penetrant like CH_4_ more than the diffusion of a smaller one like CO_2_. It can therefore be inferred that subtle changes in pore collapse lead to a significant drop in CH_4_ permeability. This results in a rise in diffusion selectivity through aging. An extreme heating protocol (longer soak time, higher pyrolysis temperature) may also cause a severe shrinkage of the pores. Additionally, a decrease in permeability could be due to the collapse of unconnected graphitic domains, which form micropores. In situ formation of copper nanoparticles in the 40% (*w*/*w*) MOP-18/BTDA-BAF could impede the collapse of the micropores and subsequently preserve the ultramicropores. Thus, the resulting Cu-pillared CMSMs would be resilient to physical aging.

**Figure 9:**
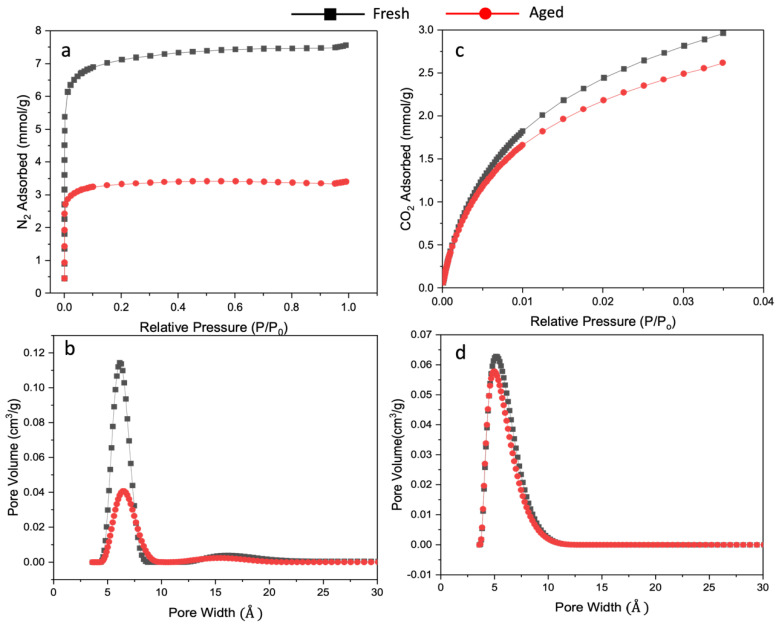
(**a**,**c**) N_2_ and CO_2_ adsorption and (**b**,**d**) related pore size distribution of CMSMs from BTDA-BAF based on 2D-NLDFT. Fresh (black) and hyperaged (red).

**Figure 10:**
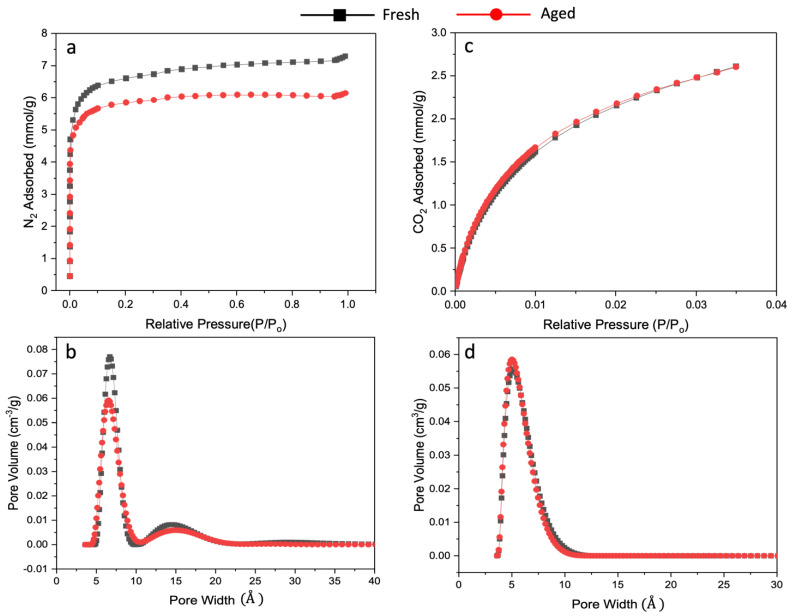
(**a**,**c**) N_2_ and CO_2_ adsorption, (**b**,**d**) related pore size distribution of fresh (black) and hyperaged (red) for 40% (*w*/*w*) MOP-18/BTDA-BAF CMSM from 2D-NLDFT.

Analysis of the nitrogen and carbon dioxide adsorption isotherms of the fresh and the hyperaged (aging in vacuum at 150 °C for 7 d) CMSMs provides insight into how the diffusivity and solubility affect the gas separation performance of the CMSMs. Expressing adsorbed gas as mmol/g and plotting the values against relative pressure (Figure 9), the aged CMSM from the pure polymer loses 55% of its N_2_ sorption capacity at P/P_0_ = 0.9 after hyperaging (at P/P_0_ = 0.9, fresh BTDA-BAF N_2_ = 7.48 mmol/g, aged BTDA-BAF = 3.35 mmol/g). This is a significant reduction in sorption capacity. From the shape of the isotherm, it can be concluded that a substantial amount of micropores collapsed along with most of the mesopores since the isotherm for hyperaged BTDA-BAF flattens above P/P_0_ = 0.2 (Figure 9). In contrast, for the 40% (*w*/*w*) MOP-18/BTDA-BAF-derived CMSMs, at the same P/P_0_ of 0.9, the hyperaged CMSM loses only 15% of its N_2_ sorption capacity (at P/P_0_ = 0.9, fresh 40% (*w*/*w*) MOP-18/BTDA-BAF N_2_ = 7.14 mmol/g, aged 40% (*w*/*w*) MOP-18/BTDA-BAF N_2_ = 6.05 mmol/g) (Figure 10). This indicates that the pillaring prevented the collapse of the majority of the micropores and some of the mesopores.

Figure 10c also shows that the CO_2_ adsorption in the CMSMs has not yet reached saturation at P/P_0_ = 0.035. If the CMSMs derived from 40% (*w*/*w*) MOP-18/BTDA-BAF had enhanced CO_2_ sorption capacities, then the amount of CO_2_ adsorbed up to P/P_0_ = 0.035 would have been significantly higher than that of N_2_ at the same P/P_0_ of 0.035. This is because CO_2_ is a much more condensable gas than N_2_, yet more than two times the amount of N_2_ is adsorbed compared to the amount of CO_2_ at P/P_0_ = 0.035. Moreover, Figure 10 shows that both the fresh and hyperaged 40% (*w*/*w*) MOP-18/BTDA-BAF CMSMs have identical CO_2_ adsorption capacities in the micropore regions and are like those of the fresh and hyperaged BTDA-BAF CMSMs, suggesting that the CMSMs derived from 40% (*w*/*w*) MOP-18/BTDA-BAF do not have increased adsorption capacity for CO_2_. Since gas solubility in the micropore region is more sensitive to the chemical environment of the pores, the number of adsorbed molecules in these pores depends mainly on the gas–adsorbent interactions rather than on the filling of the pore volume, as is the case in the mesopore region. If the CMSMs from 40% (*w*/*w*) MOP-18/BTDA-BAF had enhanced affinity for CO_2_, it should have been reflected in the isotherm as a more considerable amount of CO_2_ adsorbed by this CMSM than by the CMSM from BTDA-BAF alone. This is not observed in the isotherms shown in Figure 10. For this reason, increased solubility could mostly be disregarded as the driving force for enhanced CO_2_/CH_4_ selectivity in those CMSMs.

Figure 9b,d and Figure 10b,d illustrate the changes in pore size distribution for the non-pillared and Cu-pillared CMSM before and after aging, respectively. Nitrogen adsorption isotherms were used to calculate the surface area of the fresh and aged non-pillared and Cu-pillared CMSMs. The surface area decreased from 533 m^2^/g to 249 m^2^/g in non-pillared CMSMs, while the surface area of Cu-pillared CMSM remained essentially constant at 499 m^2^/g.

Pore collapses result in the densification of the CMSM into a tightly sintered carbon structure hindering the diffusion of N_2_ molecules into pores. The changes in micropores through aging cause a significant loss of permeability for the larger gas molecules [56].

The major decline in the N_2_ adsorption isotherm for non-pillared CMSM confirms the collapse of the micropores and, subsequently, of the ultramicropores upon aging, which is minor for the aged Cu-pillared sample (Figure 10). The CO_2_ adsorption, however, remains constant for the aged Cu-pillared CMSM, indicating that the number of not-collapsed ultramicropores is retained. The retention of CO_2_ adsorption and small changes in N_2_ adsorption in the 40% (*w*/*w*) MOP-18/BTDA-BAF is likely due to the evenly dispersed nonporous copper nanoparticles (CuNPs) that provide a scaffold for the pore structure in the CMSM.

**Figure 11:**
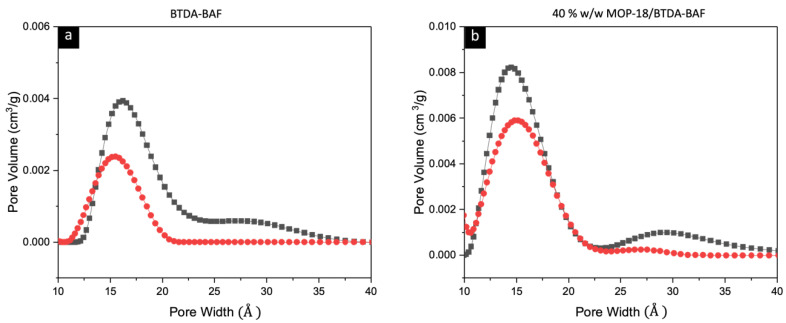
Related pore size distribution of fresh (black) and hyperaged (red) for (**a**) BTDA-BAF CMSM and (**b**) 40% (*w*/*w*) MOP-18/BTDA-BAF CMSM from 2D-NLDFT showing the changes in micropore distribution.

Pore size distribution analysis clearly shows how the micropores are affected during the hyperaging of the CMSM, which is consistent with the gas permeation properties of the CMSMs of this work. Figure 11 shows that aging of the BTDA-BAF CMSM causes the micropores to shift to a smaller pore size region, which increases the selectivity of the CMSM. This effect is not seen in the hyperaged Cu-pillared carbon membranes.

### 4.6. Gas Permeability

#### 4.6.1. Pure Gas Permselectivity of CMSM

Table 2 and Table 3 show the permeability and selectivity of pure CO_2_ and CH_4_ in fresh and aged CMSMs. Two separate membranes were tested for each sample, and the average permeability and standard deviation were calculated. Interestingly, the permeability of CO_2_ increased by incorporating MOP-18 into the BTDA-BAF polymer. This can be attributed to the higher diffusivity of CO_2_ in the 40% (*w*/*w*) MOP-18/BTDA-BAF CMSM that retains most of its pore structure compared to the BTDA-BAF CMSM. Since CO_2_ has a smaller kinetic diameter (3.3 Å) than CH_4_ (3.8 Å), it is reasonable to explain the higher permeability of CO_2_ based on this physical property. From these observations, it can be concluded that diffusion selectivity, which results from the size difference in the gas molecule, is consistent with an increase in the selectivity of the Cu-pillared carbon membrane due to the reduction in diffusivity of CH_4_ in the ultramicropores. Table 2 shows the diffusivity selectivity of gases for both carbon membranes.

Table 3 shows two separate carbon membranes’ average pure gas permeability and selectivity. Aged non-pillared CMSM showed a 29% decrease in CO_2_ permeability, while for the Cu-pillared CMSM, the permeability decreased by only 11%. The same trend has been seen for the CH_4_ permeability, indicating that physical aging affected the non-pillared CMSM much more. The aged non-pillared CMSM displayed a 56% decrease in CH_4_ permeability, while the pillared CMSM decreased by only 17.5%.

**Figure 12:**
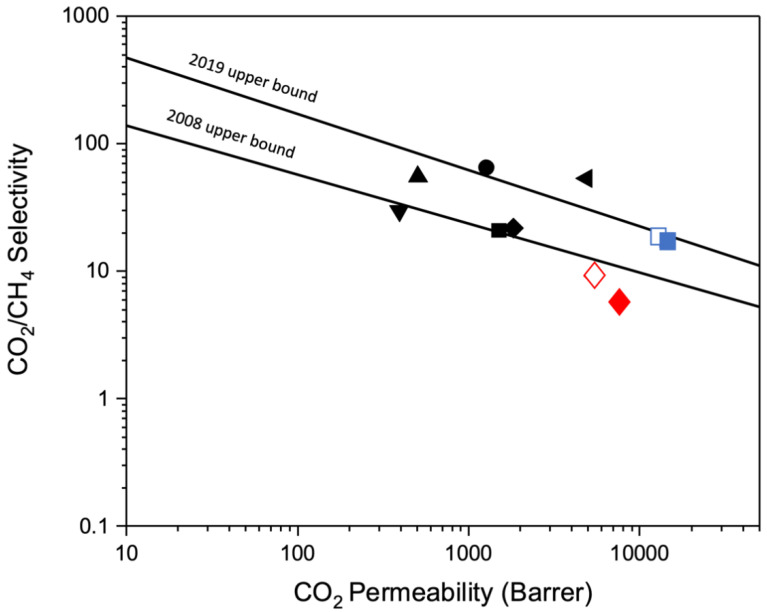
Plot of CO_2_/CH_4_ ideal selectivities versus CO_2_ permeability for fresh and aged BTDA-BAF and 40% (*w*/*w*) MOP-18/BTDA-BAF-derived CMSMs from this work and CMSMs reported in the literature [57]. CMSMs: TCM 475 (3 h) (▼), Matrimid 550 °C-2 h (●), PBI/PI (▲), SBFDA-DMN-500 (■), CM-P84-550 (♦), 6FDA/BPDA-DAM (◄), Fresh BTDA-BAF (♦) Aged BTDA-BAF (◊), Fresh 40% *w*/*w* MOP-18/BTDA-BAF(■) and Aged 40% *w*/*w* MOP-18/BTDA-BAF (□) [4,22,58,59,60,61,62].

Figure 12 shows a Robeson plot [57] for CO_2_/CH_4_ for the fresh and aged BTDA-BAF, and 40% (*w*/*w*) MOP-18/BTDA-BAF-derived CMSMs. The contorted bulky diamine in the precursor imparted high permeability in the resulting CMSMs. Moreover, the Cu-pillared CMSM performed at the 2019 upper bound, showing a permselectivity that would be desirable for gas separation.

#### 4.6.2. Stability of CMSMs to Aging

**Figure 13:**
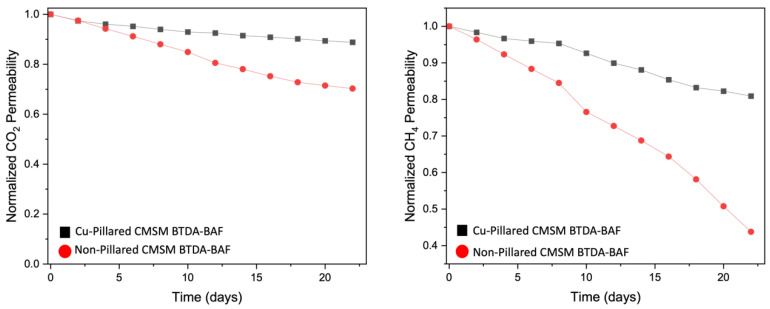
Plot of normalized CO_2_ and CH_4_ permeability versus time at 2 atm and 35 °C for non-pillared and Cu-pillared CMSMs from BTDA-BAF.

Aging studies of the pristine BTDA-BAF and 40% (*w*/*w*) MOP-18/BTDA-BAF-derived CMSMs are shown in Figure 13. CO_2_ permeability for the non-pillared CMSMs decreases over time but much less for the Cu-pillared CMSMs. This trend is even more dramatic for CH_4_ permeability for both CMSMs. Physical aging causes a decline in permeability due to pore collapses and is more pronounced for the gases with larger kinetic diameters like CH_4_.

**Figure 14:**
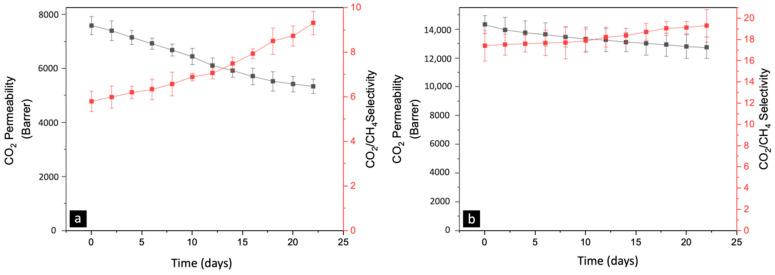
CO_2_ permeability and CO_2_/CH_4_ selectivity versus time for (**a**) BTDA-BAF CMSMs and (**b**) 40% *w*/*w* MOP-18/ BTDA-BAF CMSMs carbonized at 550-0 h.

CO_2_ permeability and CO_2_/CH_4_ selectivity versus time for both systems are shown in Figure 14. The permeability of CO_2_ is constantly declining, while CO_2_/CH_4_ selectivity is increasing for pristine BTDA-BAF CMSM. The changes in permeability and selectivity are more gradual in Cu-pillared BTDA-BAF CMSMs.

## 5. Conclusions

In this study, CMSMs derived from BTDA-BAF polyimide precursor were prepared. CMSMs from the pyrolysis of BTDA-BAF showed higher CO_2_ permeability than the CMSMs from Matrimid due to the bulky aromatic diamine group in the polyimide structure of the precursor, which was predicted to impart higher FFV. Pillaring the CMSMs with copper nanoparticles derived from the pyrolysis of MOP-18 in the 40% (*w*/*w*) MOP-18/BTDA-BAF MMMs reduced the physical aging when comparing to the non-pillared CMSM. In situ formation of copper nanoparticles smaller than 20 Å through pyrolysis of the MMM preserved the micropores of the CMSMs from collapsing. Consequently, the collapse of the ultramicropores is impeded by maintaining the micropore structure. The bigger nanoparticles that could fit on the mesopores reduce the diffusion of large gases like CH_4_ more than for the smaller gases like CO_2_. This is reason the selectivity increased in the Cu-pillared carbon membranes. It can be concluded then that the higher selectivity results from the higher diffusivity of small gases in the pillared carbon membrane.

TGA-MS showed that the MOP-18 linker’s fragments start to decompose around 380 °C, which is close to the glass transition of the BTDA-BAF (384 °C). It is hypothesized that the breakage of the linker of MOP-18 close to the T_g_ of the polymer provides more mesopores that result in high permeability for the resulting CMSM.

The results also show that pillared CMSMs exhibit permselectivities at the 2019 Robeson upper bound by retaining their selectivity and permeability upon aging, a desirable goal for achieving commercial viability.

Future work could involve the testing of other pillaring and aging stabilization materials as well as strategies for improving the control of the size distribution of the pillars. Parallel to this work, a more rigorous gas permeation testing for extended periods of up to a year may be needed to fully assess the stability of the CMSMs pillared with metal nanoparticles.

## Figures and Tables

**Table 1:** I_D_/I_G_ ratio, corresponding L_a_ values, and I_D1_/I_D2_ for BTDA-BAF and 40% (*w*/*w*) MOP-18/BTDA-BAF pyrolyzed at 550 °C-0 h.

CMSM	I_D_/I_G_	L_a_ (Å)	I_G_/I_D2_
BTDA-BAF	1.02	43.13	2.57
40% (*w*/*w*) MOP-18/BTDA-BAF	0.92	47.82	4.03

**Table 2:** Diffusivity coefficient of CO_2_ and CH_4_ in carbon membranes.

CMSM	D (cm^2^ s^−1^) 10^−8^ CO_2_	D (cm^2^ s^−1^) 10^−8^ CH_4_	α _D_
BTDA-BAF	10.8	7.3	1.48
40% *w*/*w* BTDA-BAF	12.6	4.8	2.62

**Table 3:** Pure gas permeabilities in (Barrer) of fresh and aged CMSM at 35 °C and 2 atm.

CMS Membrane	P-CO_2_	PCH_4_	*α* (CO_2_/CH_4_)
Fresh BTDA-BAF	7593 ± 339	1309 ± 90	5.8 ± 0.5
Aged BTDA-BAF	5337 ± 263	573 ± 42	9.3 ± 1
Fresh 40% (*w*/*w*) MOP-18/BTDA-BAF	14,332 ± 608	814 ± 55	17.6 ± 1
Aged 40% (*w*/*w*) MOP-18/BTDA-BAF	12,729 ± 743	659 ± 39	19.3 ± 2

## Data Availability

The data presented in this study are available on request from the corresponding author.

## Supplementary Materials

The following supporting information can be downloaded at: https://www.mdpi.com/article/10.3390/nano13162291/s1, Figure S1. The structure of carbon membrane of the other literatures. Figure S2. ^1^H NMR spectra of the BTDA-BAF. Figure S3. Optical image of (a) BTDA-BAF and (b) 40% *w*/*w* MOP-18/BTDA-BAF polymers. Figure S4. SEM images of (a,b) freeze fractured polymer cross sections of 40% *w*/*w* MOP-18/BTDA-BAF show the membrane’s thickness and homogenous distribution of MOP-18 (c) BTDA-BAF. Figure S5. DSC of BTDA-BAF and 40 % *w*/*w* MOP-18-BTDA-BAF showing glass transition at 384 °C and 436 °C, respectively. Table S1. Raman deconvolution for the carbon membranes. Figure S6. XPS spectra survey showing (a) 40% (*w*/*w*) MOP-18/BTDA-BAF CMSM and (b) BTDA-BAF. Table S2. Elemental percentage resulted from XPS analysis for CMSMs. Table S3. C 1s XPS analysis for CMSMs.

## References

[1] Rungta M., Wenz G.B., Zhang C., Xu L., Qiu W., Adams J.S., Koros W.J. (2017). Carbon Molecular Sieve Structure Development and Membrane Performance Relationships. Carbon.

[2] Cui L., Qiu W., Paul D.R., Koros W.J. (2011). Responses of 6FDA-Based Polyimide Thin Membranes to CO_2_ Exposure and Physical Aging as Monitored by Gas Permeability. Polymer.

[3] Zhang C. (2019). Synthesis and Characterization of Bis (Phenyl) Fluorene- Based Cardo Polyimide Membranes for H_2_/CH_4_ Separation. J. Mater. Sci..

[4] Xu R., Li L., Jin X., Hou M., He L., Lu Y., Song C., Wang T. (2019). Thermal Crosslinking of a Novel Membrane Derived from Phenolphthalein- Based Cardo Poly (Arylene Ether Ketone) to Enhance CO_2_/CH_4_ Separation Performance and Plasticization Resistance. J. Memb. Sci..

[5] Korshak V.V., Vinogradova S.V., Vygodskii Y.S. (1974). Cardo Polymers. J. Macromol. Sci. Part C.

[6] Tokuda Y., Fujisawa E., Okabayashi N., Matsumiya N., Takagi K., Mano H., Sato M. (1997). Development of Hollow Fiber Membranes for CO_2_ Separation. Energy Convers. Manag..

[7] Chenar M.P., Savoji H., Soltanieh M. (2011). Removal of Hydrogen Sulfide from Methane Using Commercial Polyphenylene Oxide and Cardo-Type Polyimide Hollow Fiber Membranes. Korean J. Chem. Eng..

[8] Xu Z., Dannenberg C. (2002). Gas Separation Properties of Polymers Containing Fluorene Moieties. Chem. Mater..

[9] Camacho-Zuniga C., Ruiz-Trevino F.A., Zolotukhin M.G., Castillo L.F., Guzman J., Chavez J., Torres G., Gileva N.G., Sedova E.A. (2006). Gas Transport Properties of New Aromatic Cardo Poly (Aryl Ether Ketone) S. J. Memb. Sci..

[10] Ghosh S., Banerjee S. (2014). Fluorinated Poly(Arylene Ether)s with Aliphatic Chain Appended Cardo Moiety: Synthesis and Gas Transport Properties. J. Memb. Sci..

[11] Kazama S., Teramoto T., Haraya K. (2002). Carbon Dioxide and Nitrogen Transport Properties of Bis (Phenyl) Fluorene-Based Cardo Polymer Membranes. J. Memb. Sci..

[12] Chenar M.P., Soltanieh M., Matsuura T., Tabe-mohammadi A., Feng C. (2006). Gas Permeation Properties of Commercial Polyphenylene Oxide and Cardo-Type Polyimide Hollow Fiber Membranes. Sep. Purif. Tech..

[13] Kazama S., Morimoto S., Tanaka S., Mano H., Yashima T., Yamada K., Haraya K. (2005). Cardo polyimide membranes for CO_2_ capture from flue gases. Greenh. Gas Control Technol. 7.

[14] Chenar M.P., Soltanieh M., Matsuura T., Tabe-mohammadi A., Khulbe K.C. (2006). The Effect of Water Vapor on the Performance of Commercial Polyphenylene Oxide and Cardo-Type Polyimide Hollow Fiber Membranes in CO_2_/CH_4_ Separation Applications. J. Memb. Sci..

[15] Yeong Y.F., Wang H., Pramoda K.P., Chung T.S. (2012). Thermal Induced Structural Rearrangement of Cardo-Copolybenzoxazole Membranes for Enhanced Gas Transport Properties. J. Memb. Sci..

[16] Lu Y., Hao J., Li L., Song J., Xiao G., Zhao H., Hu Z., Wang T. (2017). Preparation and Gas Transport Properties of Thermally Induced Rigid Membranes of Copolyimide Containing Cardo Moieties. React. Funct. Polym..

[17] Yahaya G.O., Mokhtari I., Alghannam A.A., Choi S., Maab H., Bahamdan A.A. (2018). Cardo-Type Random Co-Polyimide Membranes for High Pressure Pure and Mixed Sour Gas Feed Separations. J. Memb. Sci..

[18] Hu C., Polintan C.K., Tayo L.L., Chou S., Tsai H., Hung W., Hu C., Lee K., Lai J. (2019). The Gas Separation Performance Adjustment of Carbon Molecular Sieve Membrane Depending on the Chain Rigidity and Free Volume Characteristic of the Polymeric Precursor. Carbon.

[19] Sun H., Gao W., Zhang Y., Cao X., Bao S., Li P., Kang Z., Niu Q.J. (2020). Bis (Phenyl) Fluorene-Based Polymer of Intrinsic Microporosity/Functionalized Multi-Walled Carbon Nanotubes Mixed Matrix Membranes for Enhanced CO_2_ Separation Performance. React. Funct. Polym..

[20] Rahmani M., Kazemi A., Talebnia F., Gamali P.A. (2016). Fabrication and Characterization of Brominated Separation: Application of Response Surface Methodology (RSM). E-Polymers.

[21] Xu L., Rungta M., Koros W.J. (2011). Matrimid® Derived Carbon Molecular Sieve Hollow Fiber Membranes for Ethylene/Ethane Separation. J. Memb. Sci..

[22] Steel K.M., Koros W.J. (2005). An Investigation of the Effects of Pyrolysis Parameters on Gas Separation Properties of Carbon Materials. Carbon.

[23] Steel K.M., Koros W.J. (2003). Investigation of Porosity of Carbon Materials and Related Effects on Gas Separation Properties. Carbon.

[24] Hägg M., Lie J.O.N.A., Lindbråthen A. (2003). A Promising Alternative for Selected Industrial Applications. Ann. N. Y. Acad. Sci..

[25] Llosa Tanco M., Pacheco Tanaka D. (2016). Recent Advances on Carbon Molecular Sieve Membranes (CMSMs) and Reactors. Processes.

[26] Adams J.S., Itta A.K., Zhang C., Wenz G.B., Sanyal O., Koros W.J. (2019). New Insights into Structural Evolution in Carbon Molecular Sieve Membranes during Pyrolysis. Carbon.

[27] Lagorsse S., Magalh F.D., Mendes A. (2008). Aging Study of Carbon Molecular Sieve Membranes. J. Memb. Sci..

[28] Centeno T.A., Vilas J.L., Fuertes A.B. (2004). Effects of Phenolic Resin Pyrolysis Conditions on Carbon Membrane Performance for Gas Separation. J. Memb. Sci..

[29] Geiszler V.C., Koros W.J. (1996). Effects of Polyimide Pyrolysis Conditions on Carbon Molecular Sieve Membrane Properties. Ind. Eng. Chem. Res..

[30] Shao L., Chung T., Goh S.H., Pramoda K.P. (2005). The Effects of 1, 3-Cyclohexanebis (Methylamine) Modification on Gas Transport and Plasticization Resistance of Polyimide Membranes. J. Memb. Sci..

[31] Shao L., Liu L., Cheng S., Huang Y., Ma J. (2008). Comparison of Diamino Cross-Linking in Different Polyimide Solutions and Membranes by Precipitation Observation and Gas Transport. J. Memb. Sci..

[32] Wind J.D., Paul D.R., Koros W.J. (2004). Natural Gas Permeation in Polyimide Membranes. J. Memb. Sci..

[33] Wijenayake S.N., Panapitiya N.P., Nguyen C.N., Huang Y., Balkus K.J., Musselman I.H., Ferraris J.P. (2014). Composite Membranes with a Highly Selective Polymer Skin for Hydrogen Separation. Sep. Purif. Technol..

[34] Wijenayake S.N., Panapitiya N.P., Versteeg S.H., Nguyen C.N., Goel S., Balkus K.J., Musselman I.H., Ferraris J.P. (2013). Surface Cross-Linking of ZIF-8/Polyimide Mixed Matrix Membranes (MMMs) for Gas Separation. Ind. Eng. Chem. Res..

[35] Lin M., Xiao Y., Chung T., Toriida M., Tamai S. (2009). Enhanced Propylene/Propane Separation by Carbonaceous Membrane Derived from Poly (Aryl Ether Ketone)/Interpenetrating Network. Carbon.

[36] Vaughn J.T., Qiu W., Koros W.J., Xu L., Brayden M.K. (2021). Cross-Linked Polyimide Membranes and Carbon Molecular Sieve Hollow Fiber Membranes Made Therefrom. U.S. Patent.

[37] Karunaweera C., Musselman I.H., Balkus K.J., Ferraris J.P. (2019). Fabrication and Characterization of Aging Resistant Carbon Molecular Sieve Membranes for C_3_ Separation Using High Molecular Weight Crosslinkable. J. Memb. Sci..

[38] Ma C., Koros W.J. (2018). Physical Aging of Ester-Cross-Linked Hollow Fi Ber Membranes for Natural Gas Separations and Mitigation Thereof. J. Memb. Sci..

[39] Tamaddondar M., Foster A.B., Carta M., Gorgojo P., Mckeown N.B., Budd P.M. (2020). Mitigation of Physical Aging with Mixed Matrix Membranes Based on Cross-Linked PIM-1 Fillers and PIM-1. ACS Appl. Mater. Interfaces.

[40] Hernandez-Martinez H., Ruiz-Trevino F.A., Aguilar-vega M.J., Zolotukhin M.G., Marcial-hernandez R., Olvera L.I. (2018). Simultaneous Thermal Cross-Linking and Decomposition of Side Groups to Mitigate Physical Aging in Poly (Oxyindole Biphenylylene) Gas Separation Membranes. Ind. Eng. Chem. Res..

[41] Nakagawa H., Watanabe K., Harada Y., Miura K. (1999). Control of Micropore Formation in the Carbonized Ion Exchange Resin by Utilizing Pillar Effect. Carbon.

[42] Cosey W.K., Balkus Jr K.J., Ferraris J.P., Musselman I.H. (2021). Reduced aging in carbon molecular sieve membranes derived from PIM-1 and MOP-18. Ind. Eng. Chem. Res..

[43] Furukawa H., Kim J., Plass K.E., Yaghi O.M., Arbor A. (2006). Crystal Structure, dissolution, and deposition of a 5 nm Functionalized metal-organic great rhombicuboctahedron. J. Am Chem. Soc..

[44] Perez E.V., Balkus K.J., Ferraris J.P., Musselman I.H. (2014). Metal-Organic Polyhedra 18 Mixed-Matrix Membranes for Gas Separation. J. Memb. Sci..

[45] Reid B.D., Ruiz-trevino F.A., Musselman I.H., Balkus K.J., Ferraris J.P. (2001). Gas Permeability Properties of Polysulfone Membranes Containing the Mesoporous Molecular Sieve MCM-41. Chem. Mater..

[46] Riley S.J. (2000). Gas Permeability and Selectivity Studies of Pure Poly (3-[2, 5, 8-Trioxynonyl] Thiophene), PTONT, and PTONT/Poly (Ethylene Oxide) Polymer Blend Membranes.

[47] Cosey W.K., Balkus K.J., Ferraris J.P., Musselman I.H. (2020). Age Reduction in Highly Porous Carbon Molecular Sieve Membranes.

[48] Yeshchenko O.A., Dmitruk I.M., Alexeenko A.A. (2007). Size-Dependent Melting of Spherical Copper Nanoparticles Embedded in a Silica Matrix. Phys. Rev. B.

[49] Ferrari A.C., Robertson J. (2000). Interpretation of Raman Spectra of Disordered and Amorphous Carbon. Phys. Rev. B.

[50] Vallerot J., Bourrat X., Mouchon A., Chollon G. (2006). Quantitative Structural and Textural Assessment of Laminar Pyrocarbons through Raman Spectroscopy, Electron Diffraction and Few Other Techniques. Carbon.

[51] Tuinstra F., Koenig J.L. (1970). Raman Spectrum of Graphite. J. Chem. Phys..

[52] Qiu W., Vaughn J., Liu G., Xu L., Brayden M., Martinez M., Koros W.J. (2019). Hyperaging Tuning of a Carbon Molecular-Sieve Hollow Fiber Membrane with Extraordinary Gas-Separation Performance and Stability. Angew. Chem. Int. Ed..

[53] Parent P., Laffon C., Marhaba I., Ferry D., Regier T.Z., Ortega I.K., Chazallon B., Carpentier Y., Focsa C. (2016). Nanoscale Characterization of Aircraft Soot: A High-Resolution Transmission Electron Microscopy, Raman Spectroscopy, X-Ray Photoelectron and near-Edge X-Ray Absorption Spectroscopy Study. Carbon.

[54] Sadezky A., Muckenhuber H., Grothe H., Niessner R., Poschl U. (2005). Raman Microspectroscopy of Soot and Related Carbonaceous Materials: Spectral Analysis and Structural Information. Carbon.

[55] Hays S.S., Sanyal O., Leon N.E., Arab P., Koros W.J. (2020). Envisioned role of slit bypass pores in physical aging of carbon molecular sieve membranes. Carbon.

[56] Liu Z., Qiu W., Koros W.J. (2022). New Insights into Physical Aging-Induced Structure Evolution in Carbon Molecular Sieve Membranes. Angew. Chem. Int. Ed..

[57] Robeson L.M. (2008). The upper bound revisited. J. Memb. Sci..

[58] Perez-Francisco J.M., Santiago-García J.L., Loria-Bastarrachea M.I., Paul D.R., Freeman B.D., Aguilar-Vega M. (2020). CMS Membranes from PBI/PI Blends: Temperature Effect on Gas Transport and Separation Performance. J. Memb. Sci..

[59] Hazazi K., Ma X., Wang Y., Ogieglo W., Alhazmi A., Han Y., Pinnau I. (2019). Ultra-Selective Carbon Molecular Sieve Membranes for Natural Gas Separations Based on a Carbon-Rich Intrinsically Microporous Polyimide Precursor. J. Memb. Sci..

[60] Tin P.S., Chung T.S., Liu Y., Wang R. (2004). Separation of CO_2_/CH_4_ through Carbon Molecular Sieve Membranes Derived from P84 Polyimide. Carbon.

[61] Liu Z., Qiu W., Quan W., Koros W.J. (2023). Advanced Carbon Molecular Sieve Membranes Derived from Molecularly Engineered Cross-Linkable Copolyimide for Gas Separations. Nat. Mater..

[62] Comesaña-Gándara B., Chen J., Bezzu C.G., Carta M., Rose I., Ferrari M.C., McKeown N.B. (2019). Redefining the Robeson upper bounds for CO_2_/CH_4_ and CO_2_/N_2_ separations using a series of ultrapermeable benzotriptycene-based polymers of intrinsic microporosity. Energy Environ. Sci..

