# Supplementary material for: Pillared Carbon Membranes Derived from Cardo Polymers
Source: Nanomaterials (Basel). 2023 Aug 9;13(16):2291. doi: 10.3390/nano13162291 (PMC10457760; doi:10.3390/nano13162291)
Supplement: Supplementary file 1 [file nanomaterials-13-02291-s001.zip › nanomaterials-2537986-supplementary.pdf]

7. SUPPORTING INFORMATION

1. CM-P84-550

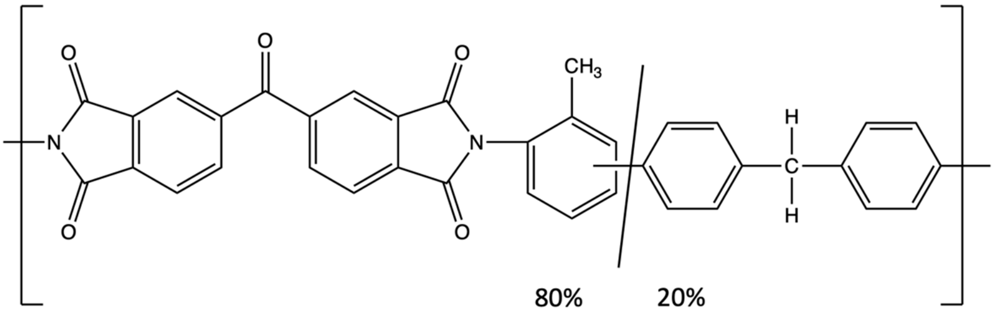

2. SBFDA-DMN-550

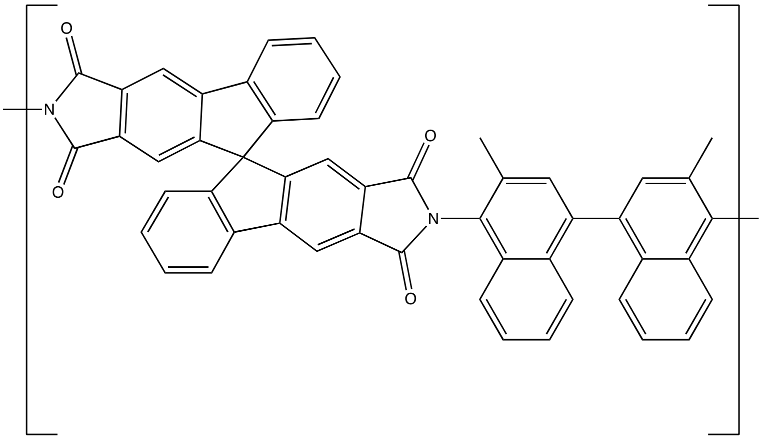

3. PI (100-600)

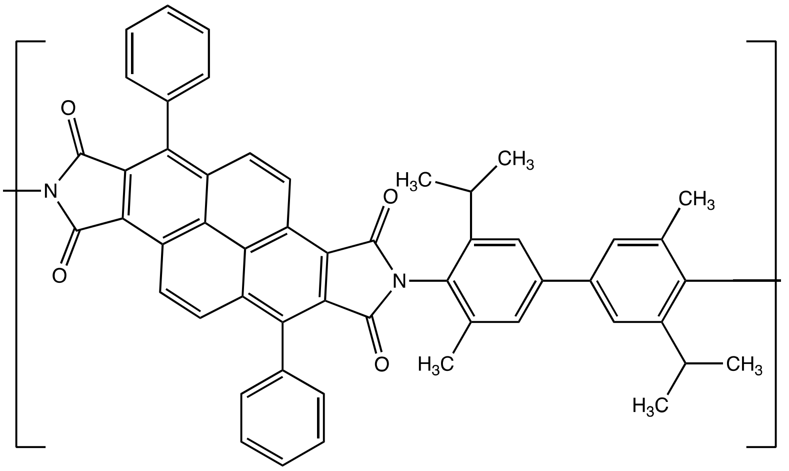

4. PEK-C (TCM-475-3h)

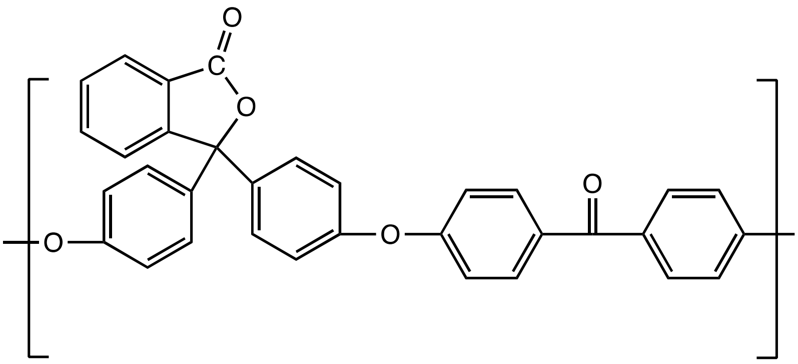

5. 6FDA/BPDA-DAM

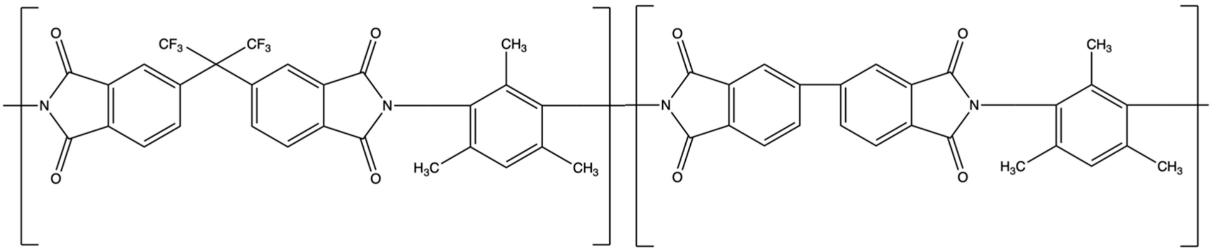

6. Matrimid

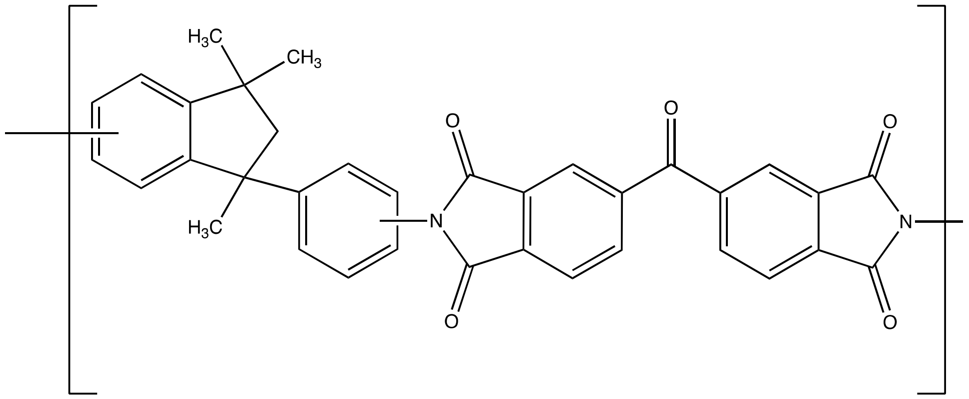

7. ODPA-FDA

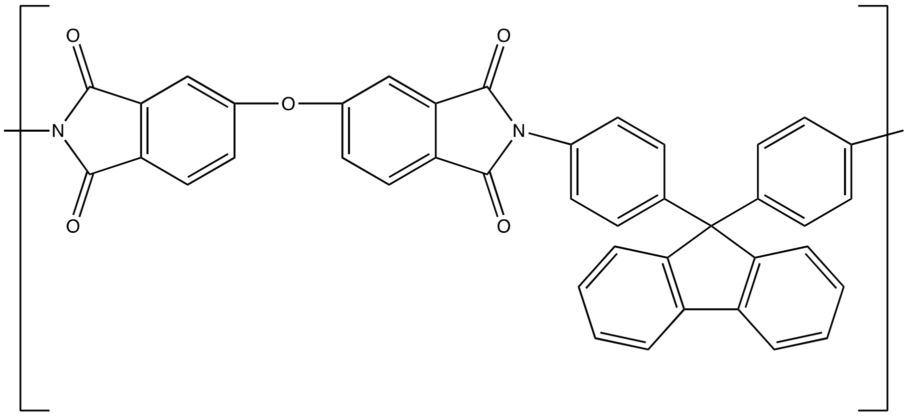

Figure S1. The structure of carbon membrane of the other literatures.

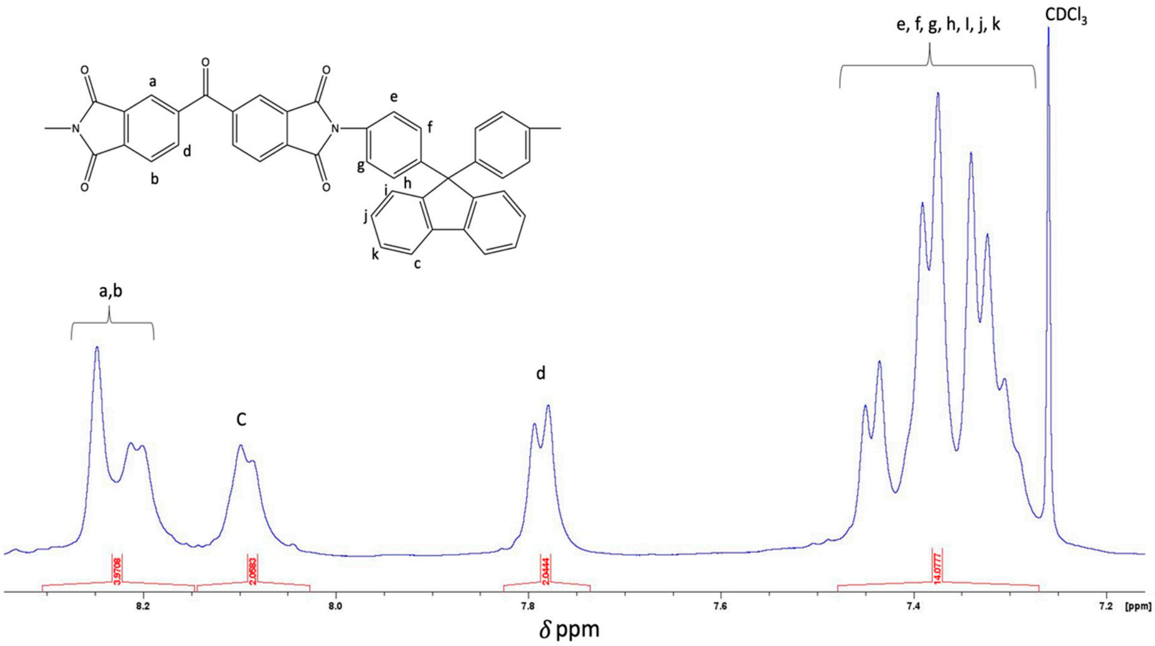

Figure S2. ^{1}H NMR spectra of the BTDA-BAF.

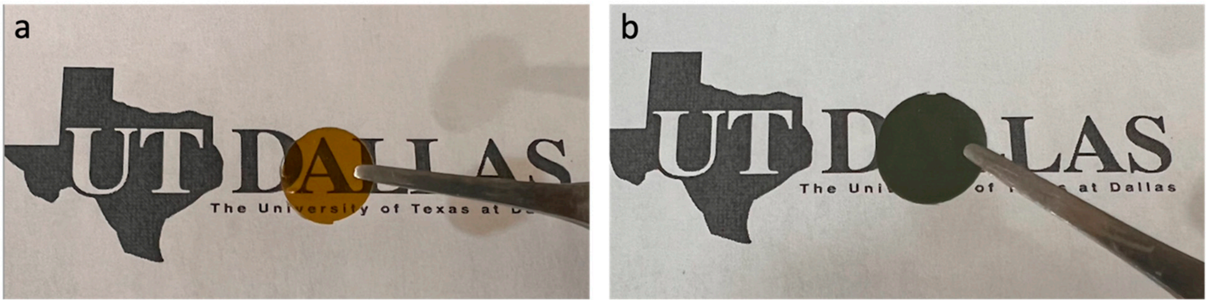

Figure S3. Optical image of a) BTDA-BAF and b) 40% w/w MOP-18/BTDA-BAF polymers.

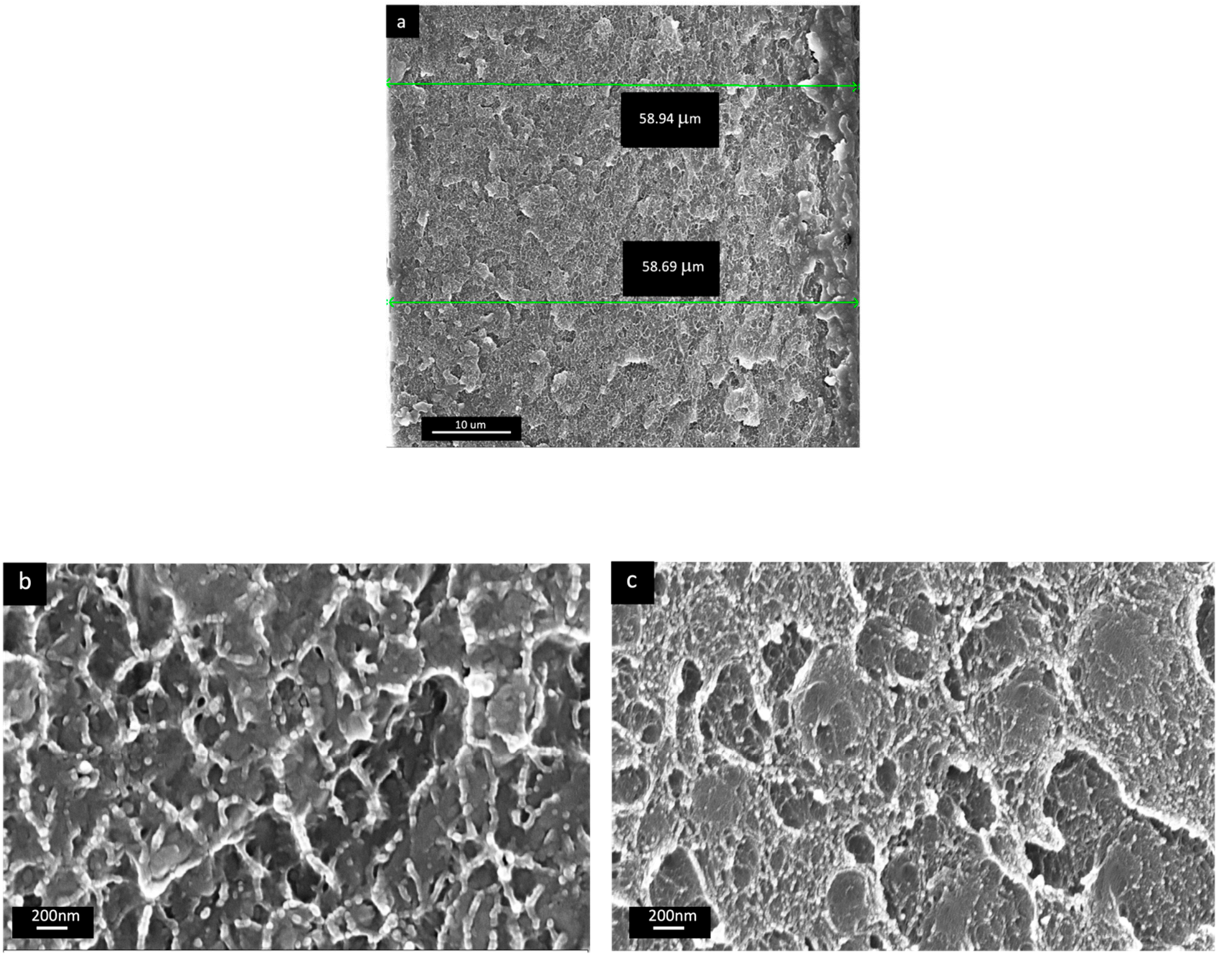

Figure S4. SEM images of a,b) freeze fractured polymer cross sections of 40% w/w MOP-18/BTDA-BAF show the membrane's thickness and homogenous distribution of MOP-18 c) BTDA-BAF.

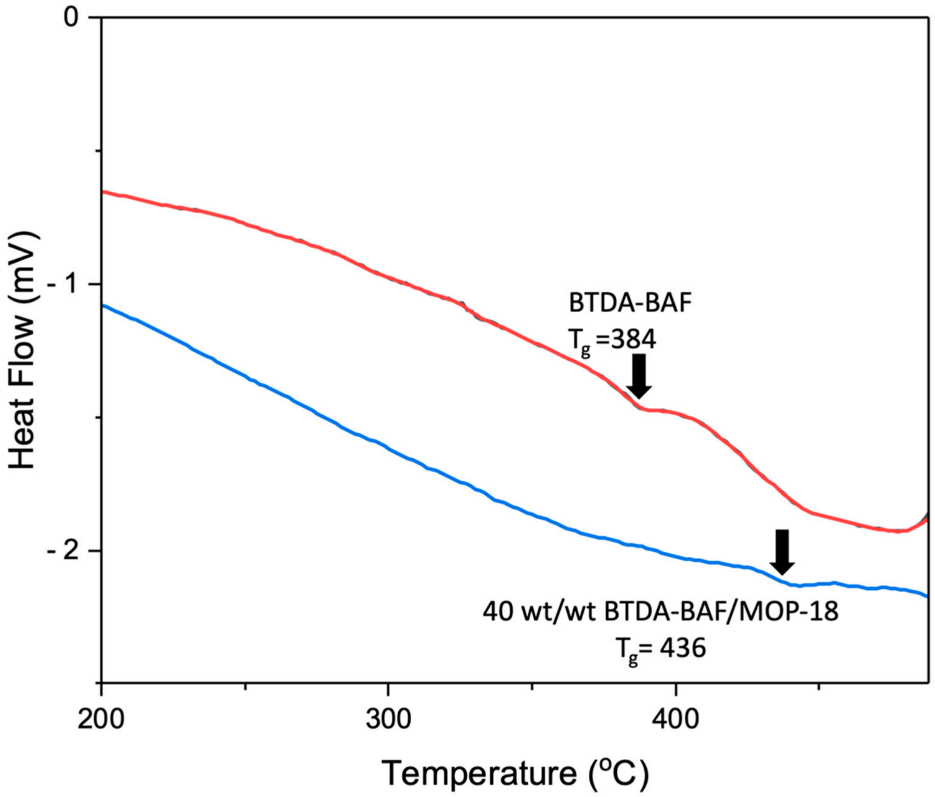

Figure S5. DSC of BTDA-BAF and 40 % w/w MOP-18-BTDA-BAF showing glass transition at 384°C and 436°C, respectively.

Table S1. Raman deconvolution for the carbon membranes.

	**CMSM**	**Fresh BTDA-BAF**	**Fresh 40 w/w MOP-18/ BTDA-BAF**
	**Position (cm^{-1})**	**Raw Height**	
**D_1**	1350	1255.38	1472.88
**D_4**	1200	261.692	289.27
**D_3**	1500	324.859	281.639
**G**	1580	1226.63	1605.78
**D_2**	1620	475.473	398.007

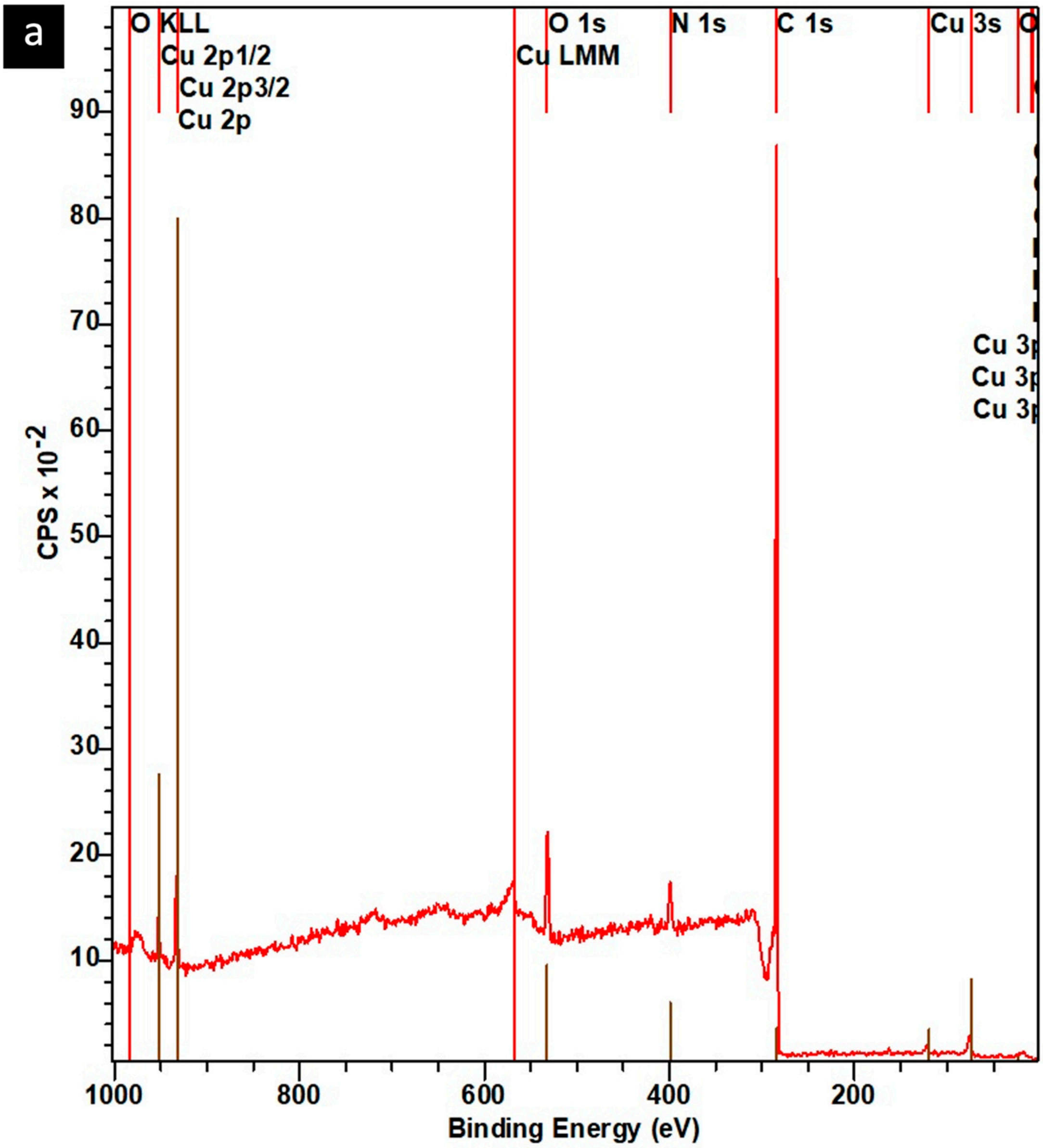
a
O KLL
Cu 2p1/2
Cu 2p3/2
Cu 2p
O 1s
Cu LMM
N 1s
C 1s
Cu 3s
Cu 3p
CPS x 10-2
90
80
70
60
50
40
30
20
10
1000
800
600
400
200
Binding Energy (eV)

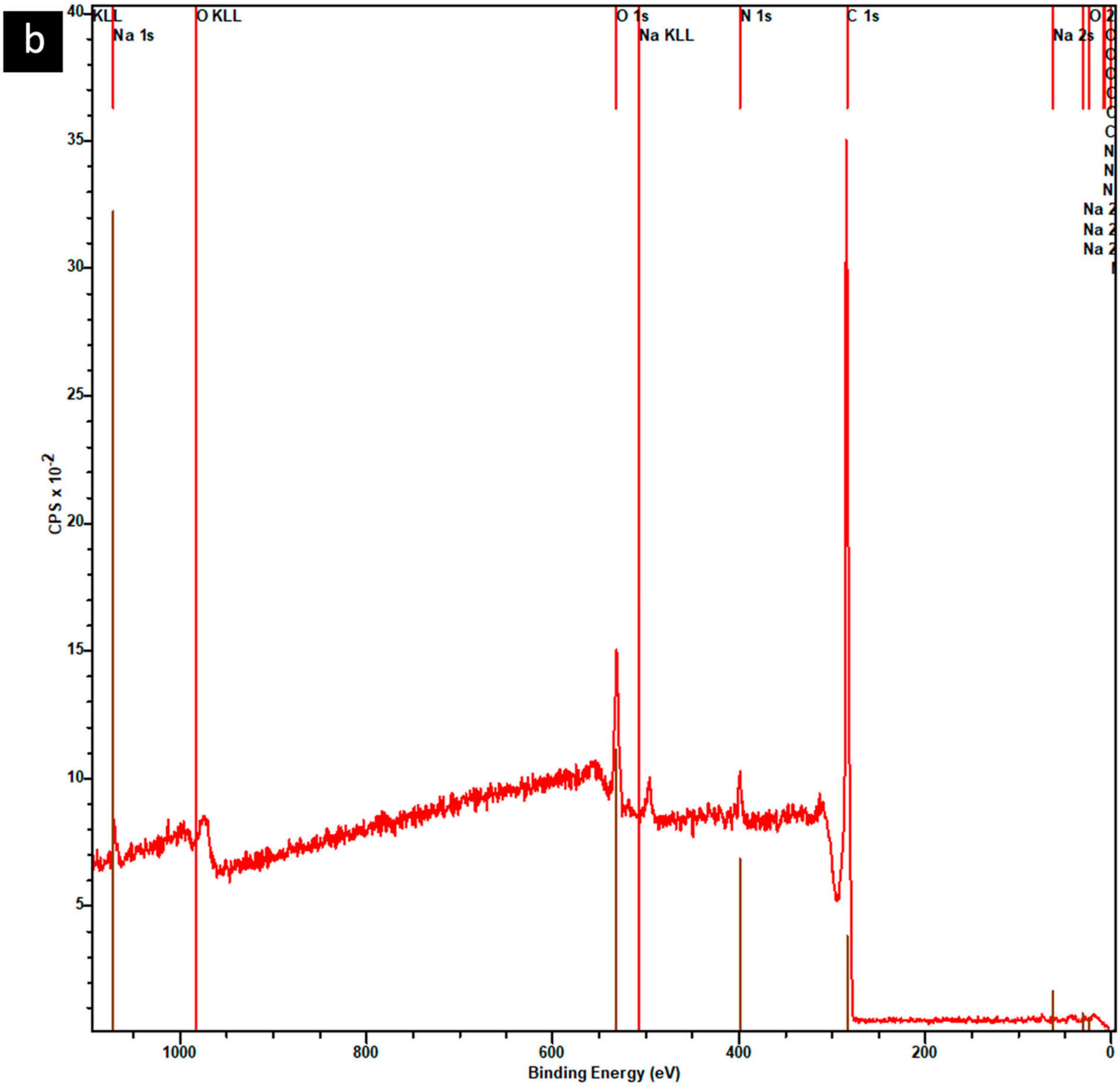

Figure S6. XPS spectra survey showing a) 40% (w/w) MOP-18/BTDA-BAF CMSM and b) BTDA-BAF.

Table S2. Elemental percentage resulted from XPS analysis for CMSMs.

**CMSM**	**BTDA-BAF**		**MOP-18/BTDA-BAF**	
Name	Position	Atom%	Position	Atom%
O 1s	531.40	3.76	531.70	3.96
C 1s	284.60	95.12	284.50	91.63
N 1s	399.80	1.12	399.30	3.30
Cu 2p	-	-	932.50	1.11

Table S3. C 1s XPS analysis for CMSMs.

**CMSM**	**BTDA-BAF**		**MOP-18/BTDA-BAF**	
	Position	Atom%	Position	Atom%
C-C	284.81	21.62	284.80	20.80
C=C	284.00	72.09	284.00	69.33
C-O	286.74	4.84	286.01	7.30
C=O	288.66	1.45	287.69	2.57
